# Dosimetric impact of phase shifts on Radixact Synchrony tracking system with patient‐specific breathing patterns

**DOI:** 10.1002/acm2.13600

**Published:** 2022-04-21

**Authors:** Mei Yan Tse, Wing Ki Claudia Chan, Tsz Ching Fok, Tin Lok Chiu, Siu Ki Yu

**Affiliations:** ^1^ Medical Physics Department Hong Kong Sanatorium and Hospital Hong Kong SAR China

**Keywords:** motion tracking, phase shifts, Radixact, Synchrony, tomotherapy

## Abstract

**Purpose:**

The Synchrony tracking system of Radixact is capable of real‐time tumor tracking by building a correlation model between external light‐emitting diodes on the patient's chest and an internal marker. A phase shift between the chest wall and a lung tumor has been reported. Hence, this study focused on evaluating the accuracy of the tracking system, especially under a patient‐specific breathing pattern with respiratory phase shifts.

**Methods:**

A phantom containing fiducial markers was placed on a moving platform. The intrinsic delivery accuracy was verified with a patient‐specific breathing pattern. Three patient‐specific breathing patterns were then implemented, for which phase shifts, *φ*, were introduced. Phase shifts with +0.3 s and +1 s were tested for dosimetric aspects, whereas ±0.3, ±0.6, and ±0.8 s shifts were used for tracking accuracy. The resultant dose distributions were analyzed by γ comparison. Dose profiles in the superior‐inferior and lateral directions were compared. Logfiles of the tracking information were extracted from the system and compared with the input breathing pattern. The root mean square (RMS) difference was used to quantify the consistency.

**Results:**

When the *φ* value was as large as 1 s, a severe inconsistency was observed. The target was significantly underdosed, down to 89% of the originally planned dose. γ analysis revealed that the failed portion was concentrated in the target region. The RMS of the tracking difference was close to 1 mm when *φ* was ±0.3 s and approximately 4 mm when *φ* was ±0.8 s. Tracking errors increased with an increase in the degree of phase shifts.

**Conclusion:**

Phase shifts between the patient chest wall and the internal target may hamper treatment delivery and jeopardize treatment using Synchrony Tracking. Hence, a larger planning target volume (PTV) may be necessary if a large phase shift is observed in a patient, especially when an external surrogate shows a lag in motion when compared with the tumor.

## 1 INTRODUCTION

Stereotactic body radiotherapy (SBRT) shows excellent local control (LC) in patients with lung^1^, ^2^, ^3^, ^4^, ^5^ and liver tumors.^6^ To reduce the uncertainty in delivering a high fractional dose to the target, motion management is necessary.^7^ Several methods, such as respiratory gating,^8^ breath‐hold method,^9^ abdominal compression,^10^ and real‐time tumor‐tracking,^11^ are used for clinical motion management. Clinical evidence has shown that some of the aforementioned techniques may cause patient discomfort and affect clinical outcomes, for example, beam gating can significantly lengthen the treatment time, whereas breath‐holding may be poorly tolerated by pulmonary‐compromised patients.^12^ Among these methods, real‐time tumor‐tracking with the CyberKnife achieves a high level of LC^11^, ^13^ and, concurrently, a reduction in the target margin.^14^

The success of CyberKnife is largely due to the Synchrony tracking system, which was introduced to the latest tomotherapy model, Radixact X9.^15^, ^16^ Three Synchrony tracking modes have been introduced, namely, irregular motion with fiducials and respiratory motion with and without fiducials. While performing respiratory tracking (e.g., for a lung tumor), three light‐emitting diode (LED) markers are attached to the patient's body to obtain the respiratory phase, and one LED is fixed on the couch as a reference position. The motion tracking system in Radixact Synchrony is similar to the system in CyberKnife. The 3D position of the target is identified by sequential monoscopic 2D kV radiography images taken at preselected gantry angles. The external surrogate position, that is, the LED, is continuously monitored using an optical camera, whereas the position of the internal markers, which may be either implanted fiducial markers or an internal anatomical structure, is detected from the radiographs. The system then builds a correlation model between the external surrogate position and the internal markers position. Throughout the treatment session, kV images are periodically acquired to continuously update the correlation model. Motion synchronization is achieved by swinging the jaw and shifting the multileaf collimator (MLC) to compensate for superior‐inferior (SI) and lateral or anterior‐posterior (AP) directional target motions, respectively.^17^, ^18^ This allows the retargeting of radiation beams to follow a moving target.

Tracking parameters are adjustable in the Synchrony system, as discussed below. Measured Δ value, which is the 2D distance between the predicted and detected fiducial or target positions, indicates the model accuracy. Rigid Body specifies the difference in the maximum fiducial pair distance between live images and the planning digitally reconstructed radiograph. In addition, Autopause Delay is a function that allows a treatment session to be paused manually or automatically when a parameter exceeds a set threshold within a certain amount of time.^15^

The uncertainty of the Synchrony system has been investigated in some studies.^15^, ^16^ One specific source of uncertainties comes from the phase difference between the external surrogate and the internal marker. Moreover, the correlation model may change due to breathing and muscle relaxation. A previous study^19^ has shown a range of phase shifts between the lung tumor and the motion of the chest wall. The phase shift was found to originate from the lag time between diaphragm‐driven respiration and the motion of the target. When performing robotic radiosurgery, the American Association of Physicists in Medicine (AAPM) Task Group 135 (TG‐135)^20^ suggests running a Synchrony end‐to‐end test with at least a 20° phase shift annually. Hence, such an end‐to‐end test should also be implemented with the similar Synchrony system on the Radixact.

In this study, we aimed to (a) analyse the ability of proactive beam synchronization of Radixact system using a patient‐specific delivery quality assurance (DQA) plan, (b) investigate the dosimetric aspects of respiratory phase shifts, and (c) evaluate the tracking accuracy between the Synchrony‐predicted target motion and the instructed motion traces when there is a phase shift between respiratory and tumor motion.

## 2 MATERIALS AND METHODS

### 2.1 Acquisition of patient data and computed tomotherapy simulation

Consent from two radiation oncologists was obtained before using patient data in this study. Patients were retrospectively considered for inclusion in the study if they were (a) treated by two referral oncologists, (b) diagnosed with lung cancer or lung metastasis, (c) underwent four‐dimensional computed tomography (4DCT) during radiation therapy simulation, and (d) completed treatment with the helical tomotherapy technique at the institution from 2018 to 2020. Patients were excluded if they had large tumor motions or large tumor sizes that would not be suitable for treatment using the Radixact Synchrony.

4DCT was performed in a CT simulator (SOMATOM Definition Edge, Siemens), coupled with real‐time position management (RPM) System (Varian, Palo Alto, CA, USA) prior to the treatment. By placing a plastic box with six infrared markers onto the thoracic surface of selected patients, respiratory motions were captured with an infrared camera. CT images with 2‐mm slice thickness were reconstructed, and 0% to 90% respiration phases were generated. Maximum intensity projection and average intensity projection images were created from the images with 0% to 90% respiration phases. The reconstructed CT images were subsequently transferred to medical image management system (MIMS, version 6.9.3; MIM Software Inc., Beachwood, OH, USA). Upon receiving the data, all patients’ demographic data were anonymized. This study and the data collection were granted approval from the institutional research committee.

### 2.2 Treatment planning

Data of an anonymized subject named *Patient‐Thx* were retrieved and optimized under the selection of Synchrony in Accuray Precision treatment planning system (TPS) (Accuray, Sunnyvale, CA, USA). The CT structure set, including the target and organ‐at‐risk contour, CT images, dose prescriptions, and dose constraints were replicated from the original treatment plans. Table 1 shows the planning parameters. Pitch and gantry rotation parameters were consistent throughout all treatments. Four image angles 30°, 135°, 225°, and 320° were selected to ensure that the fiducials inside the phantom were visible for the entire couch travel distance. Table 2 shows the tracking parameters used in the study.

**TABLE 1:** Planning parameters in the Synchrony plan

Subject	Planning target volume (cm^3^)	Dose prescription	Field width (cm)	Treatment time (s)
*Patient‐Thx*	33.53	10 fractions, 95% of gross tumor volume receives 35 Gy	2.5	172.5

**TABLE 2:** Tracking parameters used in the Synchrony treatment session

Tracking parameters	Synchrony plan for phase shift cases
Gantry period (s)	19.9
Pitch	0.29
Actual modulation factor	2
kV imaging angles (^°^)	30, 135, 225, 320
Potential diff (mm)	10
Measured Δ (mm)	4
Rigid body (mm)	1.5
Target offset (mm)	30
Target outside jaw range threshold (%)	10
Tracking range (mm)	40
Sensitivity	Medium
Auto pause delay (s)	25
kV protocol setting (kV)	Thorax (L): 120
kV protocol setting (mAs)	1.60

### 2.3 Dynamic motion phantom

A Computerized Imaging Reference Systems, Inc. (CIRS) dynamic motion platform, Model 008PL, was used to provide dynamic motion for the phantom. The platform was set to be movable in the SI direction. The external surrogate motion, which was mechanically independent of the platform motion and programmable through the CIRS Motion Control Software, moved in the AP direction. Variations in respiratory cycles, amplitude scaling, waveforms, and phase shifts could be incorporated into the three dimensions via the motion control software.

### 2.4 Delivery validation

Dosimetric accuracy measurements were performed using a stereotactic dose verification phantom (2014; Standard Imaging Inc., Middleton, WI, USA). Four inherent fiducial markers (Gold Markers, Ø 0.8 mm diameter × 5 mm long) in the phantom were imitated as implanted fiducial markers in the patient's body for target motion tracking. The phantom consisted of interchangeable ion chamber plugs and solid water slabs, which allowed the film to be sandwiched. Consequently, absolute dose measurements and film irradiation in a water‐equivalent phantom were performed simultaneously. An Exradin A16 Ion Chamber, MicroPoint, 0.007 cc was connected to TomoElectrometer at +300 V bias voltage for absolute dose measurements. For Lung Optimized Treatment (LOT), the Stereotactic Radiosurgery (SRS) dosimetric QA slab was positioned in the middle of the phantom. Using the intrinsic diverse CT density characteristic of the SRS slab to simulate complex geometric targets, the target was tracked without fiducial markers.

GAFChromic EBT3 films (Ashland Incorporated, Wilmington, DE, USA) were used for 2D dosimetry verification. The irradiated films were examined using VeriSoft 2.0 software (PTW Freiburg, Freiburg, Germany). Coronal plane pixel values within the same designated region of interest in both calculated, and measured profiles were evaluated using MATLAB (MathWorks, Natick, MA, USA) for gamma comparison. The gamma criteria were ≤3% for dose and ≤2 mm for distance to agreement (DTA) with a 10% dose threshold (3%/2 mm/10% dose threshold). Dose profiles of the SI and lateral directions of the coronal plane of the phantom were examined relative to the maximum planned dose for various breathing motions.

#### 2.4.1 The function of tracking modalities in the Synchrony system

The function of various tracking modalities in the Synchrony system was investigated by performing the standard treatment plan (*Patient‐Thx*) without phase shifts. For this DQA plan, dosimetric measurements were acquired in the following four scenarios:

1. A phantom on a static motion platform with no Synchrony tracking (NT) (i.e., the target was not moving during treatment delivery).
2. A phantom on a moving platform and with no Synchrony tracking (NTM).
3. A phantom on a moving platform with Synchrony Fiducial Tracking with Respiratory Modeling (SYN).
4. A phantom on a moving platform with Synchrony Lung Tracking with Respiratory Modeling (LOT).

Scenario NTM and NT were introduced to pinpoint the importance of motion control. A comparison between NTM and tracking modalities (SYN and LOT) was used to determine whether the correlation model in the Synchrony system improved the delivered dose distribution. All four scenarios were measured using an identical phantom setup so that the measured dosimetric discrepancies could be entirely ascribed to the capability of the Synchrony system. Megavoltage CT image registration with 2‐mm slice thickness was performed before each delivery. This verified the target and fiducial localization to minimize inter‐fractional setup error.

**FIGURE 1:**
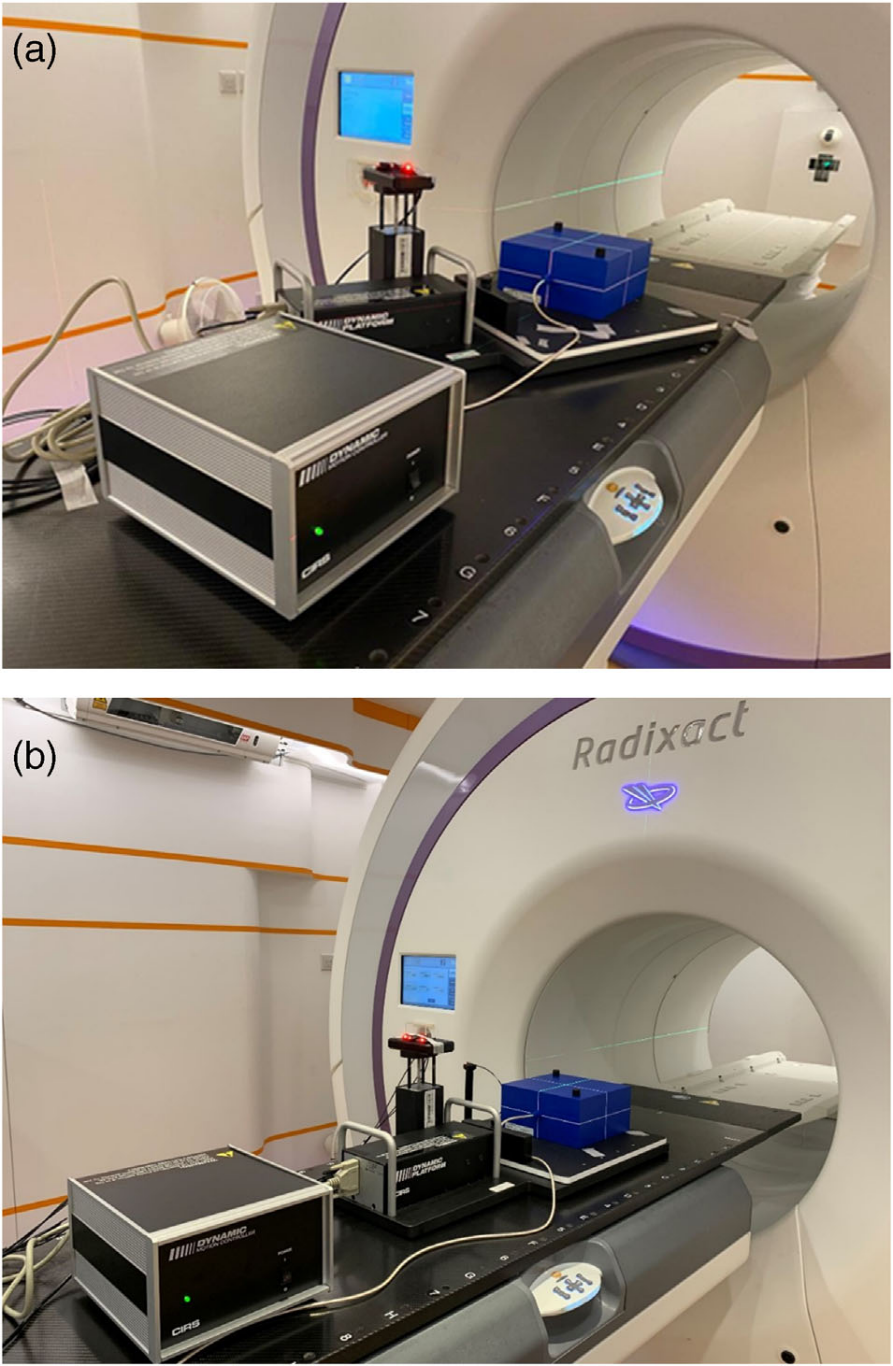
Phantom setup for dosimetric accuracy (above) and tracking accuracy (below) measurements

The measurements with the phantom setup without phase shifts are presented in Figure 1. The CIRS motion platform was set up with a yaw rotation so that its axis of motion was not aligned with the International Electrotechnical Commission (IEC‐Y) axis for simulating motion in two dimensions (IEC‐X and IEC‐Y). Here, the respiration‐induced tumor motion was measured at the three maximum edge motions of the tumor along the SI, AP, and lateral directions using the in‐built ruler tool in MIMS. The corresponding tumor motion of *Patient‐Thx* was found to be 15, 6, and 7 mm in the SI, AP, and lateral directions, respectively. Hence, an angle of 26° was calculated using the trigonometric ratio equation. The platform was rotated clockwise at an angle of 26° with respect to the IEC‐Y axis. The phantom was then positioned on the platform and aligned with the red laser. It should be noticed that motion in two dimensions (IEC‐X and IEC‐Y) allowed the testing of the synchronization of both the jaws and the MLC with the observed or predicted target motion.

#### 2.4.2 Dosimetry of phase shifts

To examine the dosimetric accuracy of the Synchrony system for phase shift cases, the standard plan *Patient‐Thx* was delivered using three patient‐specific breathing patterns. The SYN modality was selected as the tracking method. Aiming at analyzing the discrepancy attribute of the phase shifts focusing on the breathing cycle, breathing pattern, and magnitude of phase shifts, only one standard plan was utilized. This eliminated the challenges in plan quality, including various calculated dose distributions and complexities. The phantom setup with phase shifts measurements is displayed in Figure 1. The platform was set to align with IEC‐Y axis. The surrogate motion, SI directional amplitudes, and phase shifting values were input in the CIRS motion control software to drive the stereotactic phantom to move in IEC‐Y (SI) direction on the CIRS dynamic motion platform. A phase difference between the external surrogate marker and the phantom motion was introduced using the parameter *φ*. For example, *φ* = +1 s indicated that the external surrogate led the phantom SI motion by 1 s, while *φ* = ‐1 s indicated that the external surrogate lagged the phantom SI motion by 1 s. Phantom motion without a phase shift served as a reference. To simulate realistic situations, the measurement was also performed with *φ* = +0.3 s. Gamma analysis and film analysis were performed for all motions at *φ* = 0 and ±1 s, with an additional *φ* = +0.3 s for Motion C, as shown in Figure 3d, only.

### 2.5 Motion tracking validation

**FIGURE 2:**
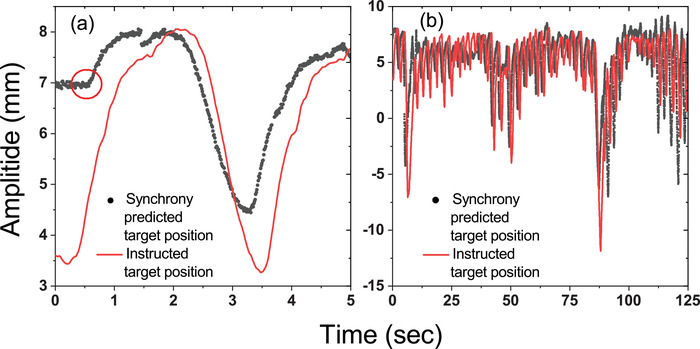
Demonstration of the comparison between the Synchrony‐predicted target position and the imported motion data, with *φ* = +0.8 s for Motion C: (a) the magnified predicted target position from machine log file with the starting point marked with the circle and (b) plots throughout the 125 s treatment times

The tracking accuracy of the standard SYN treatment plan was investigated by introducing phase shifts to three real patient motion data. The platform was set to align with IEC‐Y axis, as demonstrated in Figure 1. With the aid of accuray motion data extractor, the IEC‐Y motion of the target position predicted by the Synchrony system was extracted from machine log files. Tracking accuracy was calculated as the RMS difference between the predicted and actual motion of the phantom or target positions, as shown in Equation 1;

(1) $$\mathrm{RMS}\;=\sqrt{\frac{\Sigma_{i=1}^{N}{\left[x_{m}\left(t\right)-x_{p}\left(t\right)\right]}^{2}}{N}}\;,$$

where $x_{m}\left(t\right),\;x_{p}\left(t\right)$, and *N* refer to the machine predicted position at time t, the actual target position at time *t*, and the total number of time points, respectively. An identical time period of 125 s was set to ensure an adequate and consistent number of data points for RMS calculations when performing the analyses throughout the study. An example of fitting data points between log files generated by Synchrony system with the actual motion imported in the CIRS phantom for RMS calculations is illustrated in Figure 2. The CIRS phantom was set to move with a delay after the treatment session started. Such a delay led to a flat region that was recordable by the Synchrony system. By identifying the end of this flat region in the captured curve, correct matching between curves was achieved with a precision down to 0.01 s. Taking Motion C (*φ* = +0.8 s) as an example, Figure 2a displays the magnified predicted target position from machine log file. We defined the starting point of the Synchrony log file as the end of the flat region, and based on this starting point, we matched the data from the log file with the imported motion data at 0 s. This minimized the uncertainty of curve matching when calculating the RMS accuracy.

## 3 RESULTS

### 3.1 Respiratory motion

**FIGURE 3:**
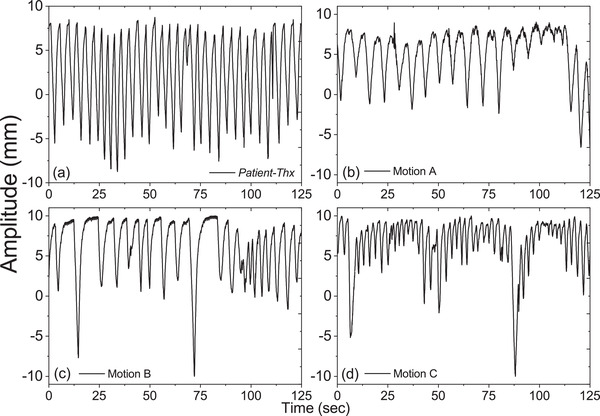
Breathing patterns of *Patient‐Thx* (a) and three sets of patient respiratory motion data (b), (c), and (d), namely Motion A, B, and C, respectively

Figure 3 shows the breathing patterns of the four selected patients, including *Patient‐Thx* and Motion A, B, and C. An external breathing amplitude of 6.39 mm with 5 s breathing period was recorded by the RPM system. Breathing cycles of Motion A and B (5 s) and Motion C (3 s) were identified as typical and short cycles, respectively. Previous studies have reported that the respiration‐induced tumor motion in patients occurs in a wide amplitude (8 to 20 mm).^21^, ^22^, ^23^, ^24^, ^25^, ^26^ Hence, we decided to apply a maximum motion amplitude of ±10 mm to simulate both the external surrogate and the SI orientation throughout the breathing cycle.

### 3.2 Dosimetry validation

#### 3.2.1 The function of tracking modalities in Synchrony system

**FIGURE 4:**
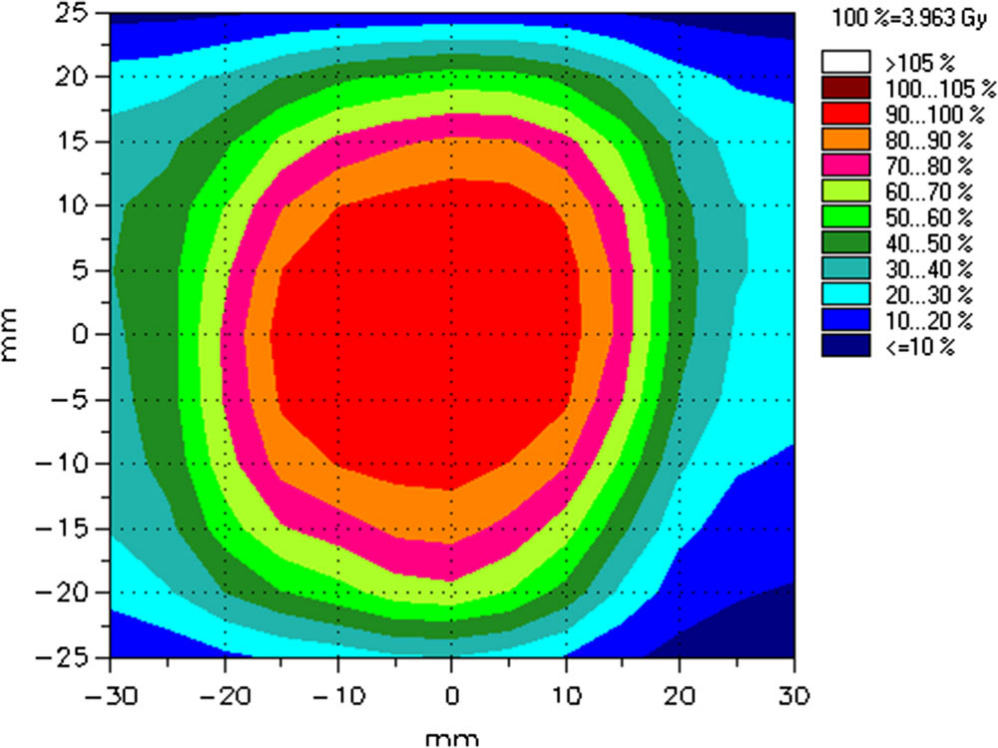
Isodose distributions of *Patient‐Thx* plan in the coronal plane

The dosimetric analyses of the four scenarios without phase shifts are presented in Table 3. An in‐house MATLAB algorithm was used to quantify the planar dose gamma distribution. The gamma passing rates for the coronal plane yielded >90% for all LOT, SYN, and NT cases, with the NT value scored close to 100%. The NTM scenario failed the gamma analysis with a value of 38.18%. Table 3 shows the absolute dose measurement for each scenario without phase shift. The differences in absolute dose measurements for all cases were within ±5%, with the NT case showing the best result of ‐0.18%. The planned isodose distribution is shown in Figure 4.

**TABLE 3:** Dosimetric analysis of four scenarios of Patient‐Thx compared to the planned dose and the absolute dose measurement for each scenario. Gamma analysis of the phantom measurement was performed with the criteria of 3%/2 mm/10% dose threshold

Scenarios	Gamma passing rates (%) of the measured doses compared with the planned dose	Difference (%) in the absolute dose measurement
LOT	97.31	+1.92
SYN	92.24	−1.07
NT	99.49	−0.18
NTM	38.18	+1.35

Abbreviation: LOT, lung optimized treatment.

#### 3.2.2 Dosimetry of phase shifts

The reference setup, that is, the phantom motion without a phase shift, showed approximately 100% gamma passing rates for all motions, as presented in Table 4. Motion C, with a shorter breathing cycle of 3 s, displayed the worst dose distribution. When applying *φ* = +1 s, the gamma passing rates (3%/2 mm/10% dose threshold) of the measured doses compared to the planned dose were down to 72.84%, 85.39%, and 56.56% for Motion A, B and C, respectively. For the ‐1 s shift, the gamma passing rates of Motion A, B, and C were 75.01%, 87.02%, and 76.60%, respectively. With a smaller +0.3 s shift, the gamma passing rate was increased to 85.43% for Motion C. Meanwhile, the delivered dose measurement results also indicated that *φ* = ±1 s caused the largest discrepancies, up to ‐10.72% and ‐7.93% in absolute dose measurements for Motion C, whereas the differences of Motion A and B were within ±5%, compared with the planned dose.

**TABLE 4:** Gamma passing rates of the measured doses compared to the planned dose and the absolute dose measurement for each breathing motion with various *φ* values. The acceptance criteria were 3%/2 mm/10% dose threshold

	Gamma passing rates (%) of the measured doses compared to the planned dose				Difference (%) in the absolute dose measurement			
φ								
motion	0 s	+0.3 s	+1 s	−1 s	0 s	+0.3 s	+1 s	−1 s
A	99.76	/	72.84	75.01	+0.08	/	+4.43	+3.71
B	99.32	/	85.39	87.02	+0.41	/	−2.76	−4.80
C	100	85.43	56.56	76.60	−0.71	−1.39	−10.72	−7.93

**FIGURE 5:**
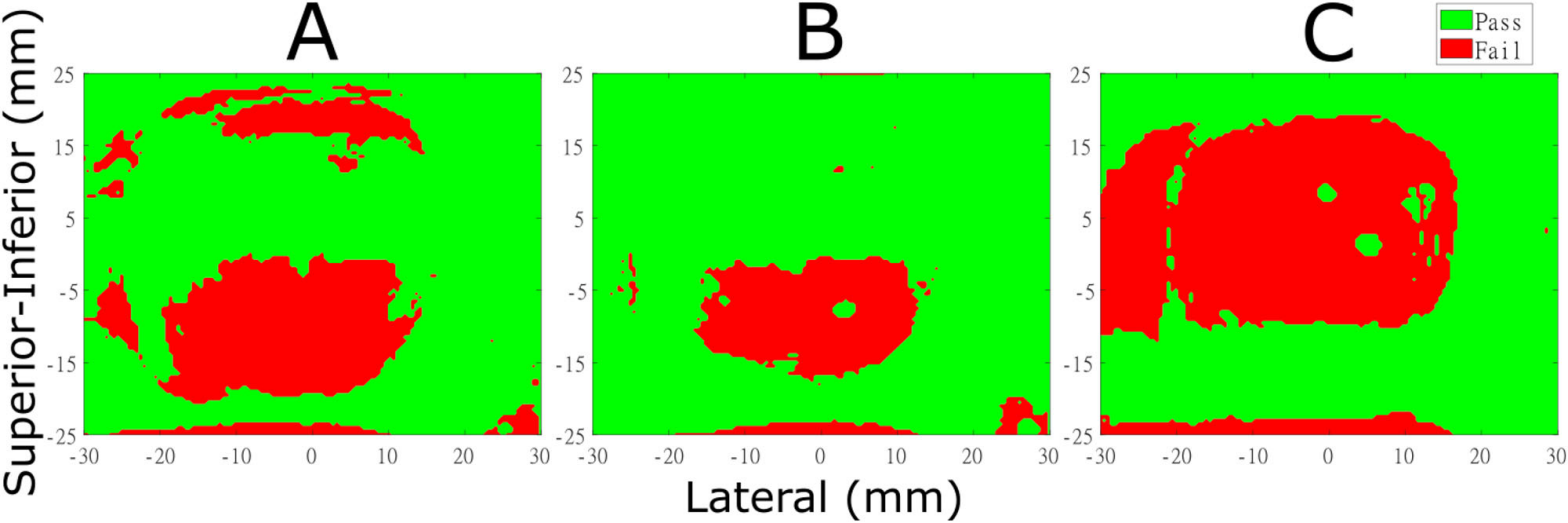
Gamma evaluation distributions in the coronal planes. Delivery was by the breathing motions with *φ* = +1 s for Motion A, B, and C; the acceptance criteria were 3%/2 mm, 10% dose threshold

Figure 5 shows the corresponding film measurements in the coronal plane when introducing a +1 s shift to Motion A, B, and C using the SYN tracking method. The failed portion was concentrated in the target region for the three motions.

**FIGURE 6:**
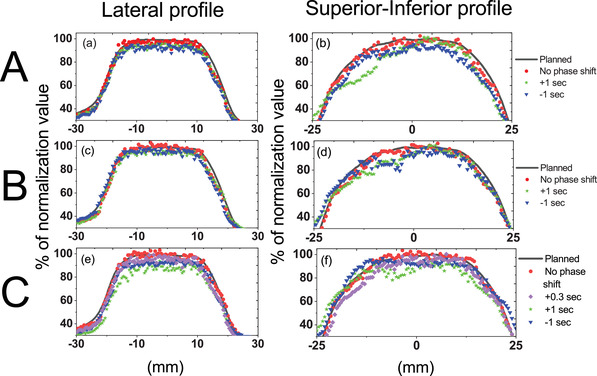
Dose profiles measured with and without phase shifts, (+0.3 and ±1 s) in the coronal plane, (a), (c), and (e), are lateral profiles of Motion A, B, and C, respectively; (b), (d), and (f), are superior‐inferior profiles of Motion A, B, and C, respectively. The planned profiles in each case were used for comparison

The lateral and SI directional dose profiles of Motion A, B, and C are shown in Figure 6. The corresponding lateral and SI directional profiles of Motion A, B, and C were taken through their origin of the IEC‐X and IEC‐Y dose planes. The delivered dose was calculated relative to the maximum planned dose. As shown in Figure 6, dose distributions were uniform and well aligned with respect to the planned dose when no phase shift was applied to Motion A, B, and C. Lateral dose profiles along the Y = 0 axis are displayed in Figure 6a–c. For Motion A and B, the maximum dose was >95% when applying a ±1 s shift. For Motion C, the dose distribution exhibited underdosing, with an increasing phase shift. The longitudinal dose profiles along the *X* = 0 axis are shown in Figure 6b,d,e. The dose profiles in the SI‐direction displayed discrepancies when phase shifts were applied. Figure 6b,d shows that there was an underdose between ‐20 to 0 mm with respect to the planned dose when applying a +1 s shift for Motion A and B. For a ‐1 s shift, an average reduction in the delivered dose of 10% was found for Motion A and B. For Motion C, the under dosage was severe between ‐15 to 10 mm, only reaching approximately 85% and 90% of the planned dose for the +1 and ‐1 s shift, respectively. The +1 s shift was shown to have more variation than the ‐1 s shift with respect to no shift, in the SI directional dose profiles.

#### 3.2.3 Motion tracking validation

**FIGURE 7:**
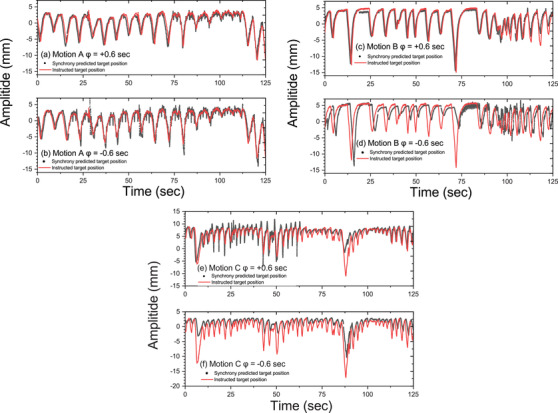
The breathing traces for comparison between the Synchrony‐predicted target position and the imported motion data with *φ* = ±0.6 s: (a) and (b) for Motion A; (c) and (d) for Motion B and (e) and (f) for Motion C

**FIGURE 8:**
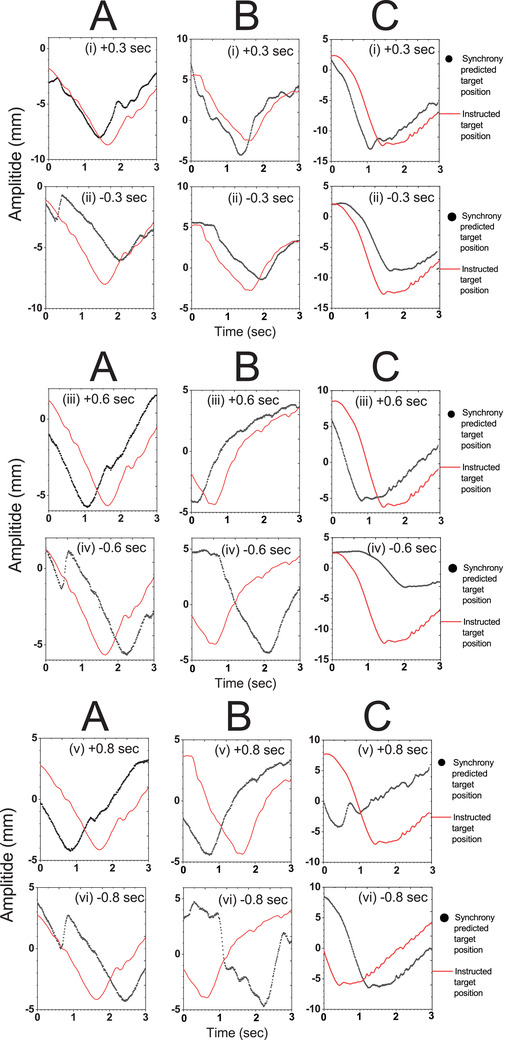
A magnification of the comparison between the Synchrony‐predicted target position and the imported motion data to indicate the actual offset: *φ* = ±0.3 s: A (i) and A (ii), B (i) and B (ii), C (i) and C (ii) for Motion A, B, and C, respectively; *φ* = ±0.6 s: A (iii) and A (iv), B (iii) and B (iv), C (iii) and C (iv) for Motion A, B, and C, respectively; *φ* = ±0.8 s: A (v) and A (vi), B (v) and B (vi), C (v) and C (vi) for Motion A, B, and C, respectively

**FIGURE 9:**
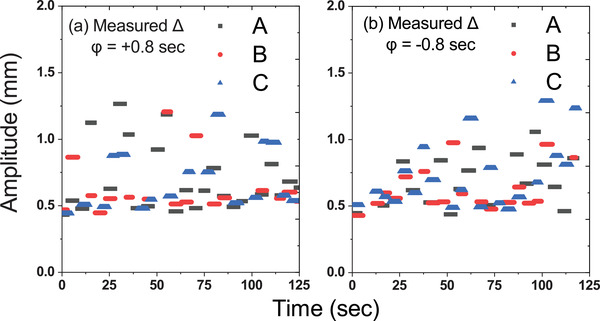
Measured Δ values during treatment delivery with, *φ* = ± 0.8 s for Motion A, B, and C

Figure 7 shows a comparison between the predicted position and the imported motion in a selected time frame for Motion A, B, and C, with *φ* = ±0.6 s. A magnified version of such a comparison depicting the peak regions of the three motions with different phase shifts is shown in Figure 8. The graphs in each case show an actual offset of the predicted target positions from the imported motion. As presented in Figures 7 and 8, Motion B, with *φ* = ‐0.6 and *φ* = ‐0.8 s, showed the greatest discrepancy from the expected offset time, while the differences between the predicted and actual motions for Motion A and C matched the magnitude of the applied phase shifts. The RMS difference between the predicted target position and the actual motion for each respiratory motion data with various phase shifts is summarized in Table 5. Notably, only Motion A attained an RMS difference below 1.5 mm when applying *φ* = ±0.3 and ±0.6 s. The overall results exhibited a trend of increasing RMS difference with greater phase shifts. The measured Δ values in real‐time throughout the treatment session are presented in Figure 9. The average measured Δ values for Motion A, B, and C were 0.70, 0.68, and 0.63 mm, respectively, with *φ* = +0.8 s and 0.62, 0.66, and 0.72 mm, respectively, with *φ* = ‐0.8 s. All of these values fell within the preset tracking limit.

**TABLE 5:** Root mean square difference between the predicted and actual target positions

Φ	+0.3 s	−0.3 s	+0.6 s	−0.6 s	+0.8 s	−0.8 s
Motion A	0.96	0.99	1.36	1.30	2.05	1.70
Motion B	1.29	1.57	1.87	4.08	4.02	4.09
Motion C	1.68	1.16	2.44	2.83	3.32	2.62

## 4 DISCUSSION

The ability of proactive beam synchronization using the Radixact system was investigated using phantom study. The gamma passing rate was satisfactory with the SYN and LOT tracking modalities when compared with the NTM modality. The results revealed that the Synchrony system, with the real‐time compensation technique, was effective at precise dose delivery during respiratory motions. These findings were consistent with those reported by Chen et al.’s study.^15^ In addition, when comparing the results for NT and NTM modalities, NTM showed the worst gamma distribution. This indicates the necessity for tumor motion control during treatment. Nevertheless, the absolute point dose measurement for NTM was +1.35%, which was comparable with that of the SYN and LOT modalities. This may be attributed to the large target size (>4 cm in diameter) for *Patient‐Thx* plan. Therefore, the influence of target motion was less due to the small ion chamber placed in the middle of the target. As expected, the gamma passing rates (3%/2 mm/10% dose threshold) were close to 100% for all motions without phase shifts in the SYN scenario (Table 4), indicating the reliability of motion tracking by the Synchrony system.

Phase shifts between respiratory and tumor motions may cause discrepancies between the delivered dose distribution and the planned dose. Based on the AAPM Task Group TG‐218 recommendations, a 3% dose difference and a 2‐mm DTA with 95% gamma passing criteria, were used in this study.^27^ Both gamma evaluations and absolute dose measurements indicated a notable dose discrepancy compared with the case when no phase shift was applied. Motion C exhibited the greatest percentage difference in the gamma passing rate, at approximately 57%, compared with Motion A and B. This finding may be attributed to the shorter breathing cycle of 3 s and the inherent irregular breathing patterns in Motion C compared with Motion A and B, which had a breathing cycle of 5 s and relatively regular and slightly irregular breathing patterns, respectively.

The International Commission on Radiation Units and Measurements^28^ recommends that the overall accuracy of the patient dose should be within ±5% of the prescription dose. The absolute dose measurements for all breathing motions with no phase shifts were within 99% of the intended dose, whereas those for Motion A and B, with a ±1 s shift, were within 95%, implying that even with a shift of 1 s, the delivered dose may still be within the clinically acceptable range. In clinical practice, tumors sometimes follow more complicated motion paths. We demonstrated an actual scenario using Motion C, with an additional +0.3 s shift. The dose distribution of the +0.3 s shift showed a greater dose conformity than that of the ±1 s shift. The large discrepancy found in the +1 s shift suggests that clinicians should be cautious in conditions where phase shifts between the patient's chest wall and the internal targets are observed and breathing cycles appear to be relatively short.

The gamma distribution results were consistent with the lateral and the SI directional dose profiles. Since the dynamic platform was set to move in the SI direction, an underdose in the SI profiles for all motions was within expectations. The phenomenon whereby Motion C exhibited reduced dose with increasing phase shift period may have occurred because Motion C itself was regarded as a short respiratory cycle, and, hence, it gave relatively unstable performance.

Previous studies have evaluated motion tracking accuracy with respiratory phase shifts between the motion of the lung tumor and the chest wall.^15^, ^22^, ^29^, ^30^, ^31^ However, those studies did not focus on patient‐specific breathing patterns. Akino et al. demonstrated the motion tracking accuracy of the CyberKnife Synchrony system using a plastic scintillator.^22^ Significant tracking errors were found for cases with a respiratory phase shift between the surrogate and target motions, which is consistent with our findings.

The AAPM Task Group TG‐135 suggests a tolerance of 1.5 mm for motion‐tracking treatments involving Synchrony.^20^ Our results indicated that motion tracking accuracy was inferior when the phase shift increased. Moreover, negative phase‐shifted periods, in general, gave larger tracking errors than positive phase‐shifted periods, that is, when the motion of the internal target led the external surrogate. This was particularly evident for the ‐0.6 s shift, which had a relatively high pattern resemblance but failed the expected offset (‐0.6 s). Although the ±0.8 s shift yielded a greater error, the shifted period was relatively large, such that it outweighed the distorted breathing pattern. This observation was consistent with the findings reported by Akino et al.^22^

The effect of modeling parameters on tracking accuracy was also investigated. The measured Δ values logged during the treatment session were extracted. The Δ values were far below the threshold of 4 mm, even with the extreme ±0.8 s shift. Furthermore, there was no interruption during treatment delivery, indicating that the target position was tracked with an appropriately correlated statistical model.

Ferris et al. concluded that the Radixact Synchrony system may not be sensitive to surrogate phase shifts from 3D respiratory motion.^16^ A possible reason for the discrepancy between their results and ours may be that they selected a regular respiratory pattern with similar target motion magnitude and period. For those cases, the Synchrony system was capable of surmising such a phase shift and building a corresponding model. On the contrary, demonstrating phase shifts using irregular breathing motions, as we did in this study, may be more practical for clinical situations. Our results indicated that the Radixact Synchrony system was still sensitive to the effect of phase relationship and may exhibit uncertainties in displacement when the patient breathes irregularly with short breaths.

Our findings show that the real‐time motion compensation techniques of the Synchrony system maintain delivery and tracking accuracy only for patients with a mild and comparable regular respiratory phase shift between the lung tumor and external chest wall motions. This may allow planning with minimal planning target volume (PTV) margin expansion. However, for tumors following more complicated motion paths or in cases with an accidentally steep motion peak in the breathing traces, discrepancies in the dose distribution and large tracking errors are expected. Keall et al. stated that the tumor location, combined with the tumor pathology, may lead to various distinct patterns in displacement, direction, and phase.^26^ In these extreme cases, it is important to note that it may be more challenging to build a correlation model, or interruptions may even be encountered during treatment delivery, which would increase the treatment time. Another approach for patients with irregular breathing traces, more frequent movements, or greater sensitivity to uncorrected motion is to acquire more image angles per rotation. Moreover, it has been suggested that tumor delineation and, consequently, the planning target margins should be investigated on a case‐by‐case basis when there is a respiratory phase shift between the target tumor and the surrogate. The consideration to reduce margins may rely greatly on the precision of the predicted algorithm and the accuracy of image acquisition. The present study did not consider different amplitudes of the SI and external surrogate breathing patterns of the patients. Tumors with various amplitudes and directions will be examined in a future study. Amplitudes may affect the prediction capability of the Synchrony system, because if there is no significant movement of the interposed tissue, the movement of the tumor may be predicted with high accuracy.

## 5 CONCLUSIONS

The real‐time motion tracking Synchrony system enabled accurate target tracking and excellent dose delivery under normal free‐breathing conditions. However, phase shifts found between patient‐external breathing and the internal target position may affect the system tracking performance with unsynchronized or falsely predicted models. The magnitude of tracking errors increased with the degree of phase shift, and the most adverse situation emerged when the internal target motion led the external surrogate. It has been suggested that tumor delineation and, consequently, the planning target margins should be investigated on a case‐by‐case basis when there is a respiratory phase shift between the lung tumor and the external chest wall motions.

## CONFLICT OF INTEREST

The authors declare that there is no conflict of interest that could be perceived as prejudicing the impartiality of the research reported.

## AUTHOR CONTRIBUTIONS

*Data acquisition, analysis, and drafting the work*: MYT. *Data acquisition, analysis, and drafting the work*: WKCC. *Data acquisition, analysis, and drafting the work*: TCF. *Data acquisition, analysis, and design of the work*: TLC. *Conception of the work*: SKY.
